# Mutation of copper binding sites on cellular prion protein abolishes its inhibitory action on NMDA receptors in mouse hippocampal neurons

**DOI:** 10.1186/s13041-021-00828-0

**Published:** 2021-07-19

**Authors:** Sun Huang, Stefanie A. Black, Junting Huang, Peter K. Stys, Gerald W. Zamponi

**Affiliations:** 1 Department of Physiology and Pharmacology, University of Calgary, Calgary, AB T2N 4N1 Canada; 2 Department of Clinical Neurosciences, University of Calgary, Calgary, AB T2N 4N1 Canada; 3 Hotchkiss Brain Institute, University of Calgary, Calgary, AB T2N 4N1 Canada; 4 Alberta Children’s Hospital Research Institute, University of Calgary, Calgary, AB T2N 4N1 Canada

**Keywords:** NMDA receptor, Cellular prion protein, AAV system, Knock-out mice, Hippocampal neurons, Whole-cell patch clamp, CNS disorders, Copper

## Abstract

We have previously reported that cellular prion protein (PrP^C^) can down-regulate NMDA receptor activity and in a copper dependent manner. Here, we employed AAV9 to introduce murine cellular prion protein into mouse hippocampal neurons in primary cultures from PrP null mice to determine the role of the six copper binding motifs located within the N-terminal domain of PrP^C^. The results demonstrate that viral expression of wild type PrP^C^ lowers NMDAR activity in PrP null mouse hippocampal neurons by reducing the magnitude of non-desensitizing currents. Elimination of the last two copper binding sites alone, or in combination with the remaining four attenuates this protective effect. Thus our data suggest that copper ion interactions with specific binding sites on PrP^C^ are critical for PrP^C^ dependent modulation of NMDA receptor function.

N-methyl-d-aspartate receptors (NMDARs) are a key class of glutamate receptors that mediates a wide range of central nervous system functions [1, 2]. Excessive NMDAR activity causes calcium toxicity and may result in neurodegeneration in disorders such as stroke and Alzheimer’s disease [3, 4]. Cellular prion protein (PrP^C^) is widely expressed in the mammalian nervous system and mediates neuroprotective functions. Previous studies revealed that PrP^C^ can physically interact with NMDARs and exert an inhibitory functional regulation [5–7]. Specifically, knockout of PrP^C^ leads to slowly desensitizing NMDAR currents across a range of glycine co-agonist concentrations, leading to tonic receptor activity that causes neurotoxicity [5–7]. Conversely, we recently explored transgenic PrP^C^ mouse models to demonstrate that overexpression of PrP^C^ downregulates NMDAR activity [8]. PrP^C^ is a known copper binding protein that interacts with these metal ions with up to attomolar affinity, with six putative copper binding sites localized within the N-terminal region of PrP^C^ [9]. Copper interactions with PrP^C^ have been shown to affect its ability to regulate NMDA receptor desensitization. For example, copper chelation by bathocuproine disulfonate (BCS) [10] results in more slowly desensitizing NMDAR currents, thus suggesting that copper ions mediate this effect via PrP^C^. Here, we tested the hypothesis that copper binding sites on PrP^C^ are required for regulation of NMDAR receptor activity.

Animal experiments were conducted with the approval of the animal care committee of the University of Calgary. Wild-type C57 mice were purchased from Charles River, PrP^C^ knockout (KO) mice and Tga20 mice (overexpressing the murine cellular prion protein) were provided by Dr. Frank Jirik. P0–P1 pups were obtained to prepare hippocampal neurons for primary culture as described by us [8]. We first performed whole cell patch clamp experiments on hippocampal pyramidal neurons from WT, PrP^C^ null and Tga20 mice at 11–13 DIV to measure NMDA currents. The external solution contained 140 mM NaCl, 5 mM KCl, 1 mM CaCl_2_, 25 mM HEPES, and 33 mM d-Glucose, (pH 7.4, NaOH), and supplemented with 15 µM 2,3-dihydroxy-6-nitro-7-sulfamoyl-benzo[f]quinoxaline, 100 μM picrotoxin, 1 μM tetrodotoxin, 500 nM CuSO_4_ (to standardize [Cu^2+^] in the external medium), and different concentrations of the NMDAR co-agonist glycine as indicated. The pipette solution contained 140 mM CsCl, 11 mM EGTA, 1 mM CaCl_2_, 2 mM MgCl_2_, 10 mM HEPES, 4 mM K_2_ATP and 0.6 mM GTP (pH 7.3, CsOH). 500 µM NMDA (Tocris Bioscience) was applied with a microperfusion system (EVH-9, Biologic Science Instruments) to achieve rapid solution exchange and activation of NMDA currents. The holding potential was − 60 mV throughout, and agonist was applied for 7 s before washout.

Figure 1a represents typical NMDA currents in hippocampal pyramidal neurons from mouse lines with different PrP^C^ expression levels in the presence of 1 μM glycine. Consistent with our previous work, NMDA currents in Tga20 neurons exhibited decreased steady state current compared to wild type. On the other hand, PrP^C^ KO leads to increased steady state current as described by us previously. This effect is quantified over a range of glycine concentrations in Fig. 1b. These data reconfirm that higher levels of PrP^C^ reduce non-desensitizing NMDA current activity whereas removal of PrP^C^ has the opposite effect. We attribute these effects primarily to alterations in glycine regulation [5]. However, we previously reported that the absence of PrP^C^ alters NMDAR subunit composition and this might contribute to changes in the decay kinetics (although we note that this subunit switch predominantly affected deactivation rather than desensitization) [7].

**Fig. 1:**
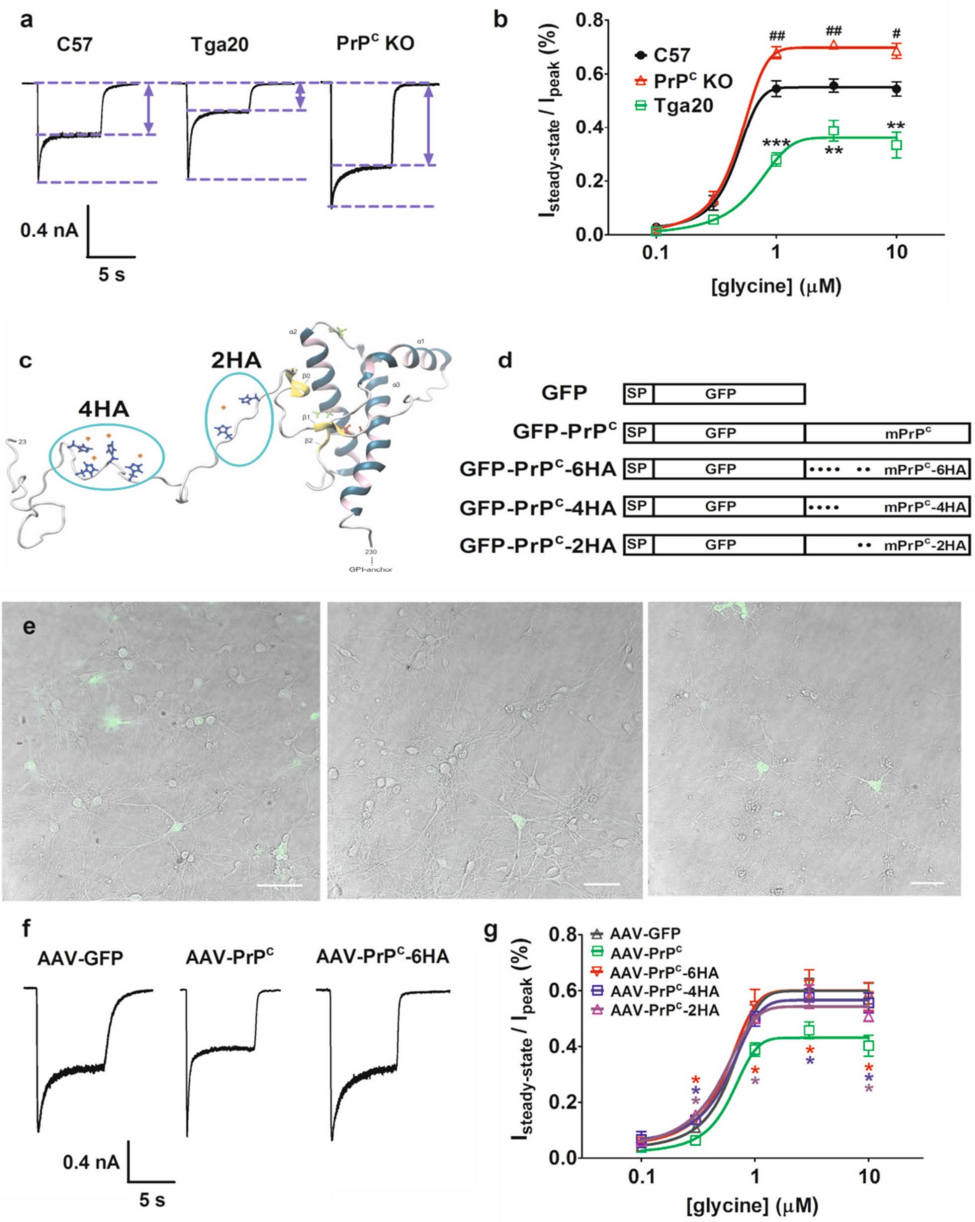
**a** NMDAR-mediated currents from hippocampal neuron cultures of PrP^c^ knock-in Tga20 mice, PrP^c^ knock-out PrP^c^ KO mice versus wild-type C57. Neurons were held at − 60 mV throughout and currents were evoked by application of 500 µM NMDA and 1 μM glycine. The dashed lines indicate baseline, steady state current, and peak current, and the arrows indicate the magnitude of the non-desentitizing (steady state) current. **b** Glycine dose response curve of the percentage of steady-state current (normalized to peak) in wild-type C57, Tga20 and PrP^c^ KO muse neurons (n = 5). Asterisks denote statistical significance for C57 vs Tga20, and number symbols indicate statistical significance between C57 and PrP^c^ KO at the 0.05, 0.1 and 0.001 levels for one, two and three symbols, respectively (one way ANOVA with Bonferroni post hoc test). **c** Structure of PrP^c^ illustrating the location of copper binding motifs in the unstructured N-terminal region (taken from [11]). **d** Illustration of constructs of recombinant AAV-GFP-PrP^c^ and the various copper site mutants. **e** Confocal images of hippocampal neurons from PrP^c^ KO mice transduced with AAV9 expressing eGFP (left), mPrP^c^ (middle), and mPrP^c^-6HA (right). Green signal reflects eGFP fluorescence and thus PrP^c^ expression. The primary hippocampal neurons were cultured for 3 days before transduction and confocal images were collected 8 days later. The dose was 1 × 10^11^ GC/ml for all constructs. Scale bar = 50 µm. **f** Representative traces of NMDAR currents from hippocampal neurons of PrP^c^ KO mice infected with AAV-GFP, AAV-GFP-PrP^c^, and AAV-GFP-PrP^c^-6HA. Neurons were held at − 60 mV throughout and currents were evoked by application of 500 µM NMDA and 1 μM glycine **g.** Glycine dose response curve of the percentage of steady-state current (normalized to peak) in neurons from PrP^c﻿^ KO mice transduced with AAV-GFP, AAV-GFP-PrP^c^, AAV-GFP-PrP^c^-6HA, AAV-GFP-PrP^c^-4HA and AAV-GFP-PrP^c^-2HA (n = 6 for AAV-GFP and AAV-GFP-PrP^c^, n = 5 for AAV-GFP-PrP^c^-6HA, AAV-GFP-PrP^c^-4HA and AAV-GFP-PrP^c^-2HA). Asterisks refer to statistical difference relative to AAV-GFP-PrP﻿^﻿c^ (One way ANOVA with Tamhane-Dunnett's Test [13]), with colour of the asterisks corresponding to the colour denoting the various PrP^﻿c^ mutant constructs

The unstructured N-terminus of PrP^C^ contains copper binding sites formed by four histidines within the octapeptide repeats closer to the N-terminus and two histidines outside of the octapeptide repeats closer to the C-terminal end of this region Fig. 1c [11]. To test the roles of these copper sites, we packaged cDNA of murine cellular prion protein, along with the signal peptide (SP) and green fluorescent protein (GFP) into AAV9 vectors to generate AAV-PrP^C^ constructs (Fig. 1d). To monitor putative effects of GFP overexpression, we also created an AAV-GFP construct. Partial or full ablation of copper binding domains was achieved by replacing the two histidines outside the octarepeat, four histidines inside the octarepeat, or all six key histidines with alanines. We then infected PrP null mouse hippocampal cultures with AAV-GFP, wild type GFP-PrP^C^, or the three different copper mutant AAV constructs. Figure 1e illustrates hippocampal neurons 8 days post infection, with robust GFP expression being evident. We then performed whole cell patch clamp experiments as outlined above, by selectively patching on to the GFP positive neurons. Figure 1f illustrates NMDAR currents in pyramidal neurons from PrP^C^ null mouse neurons transduced with AAV-GFP, AAV-GFP-PrP^C^, or a mutant in which all six copper binding sites had been abolished (AAV-GFP-PrP^C^-6HA). With the reintroduction of PrP^C^, NMDAR current desensitization kinetics were normalized to levels similar to those seen in WT mouse neurons **(**compare Fig. 1a and f). This rescue effect was abolished when the six copper binding sites were mutated (Fig. 1f). We further tested two mutants in which either the first four or the last two histidines were mutated. Figure 1g summarizes aggregate data for NMDAR desensitization over a range of glycine concentrations for the various constructs, illustrating the differing NMDAR desensitization kinetics. Removal of all six (AAV-GFP-PrP^C^-6HA), the final two (AAV-GFP-PrP^C^-2HA) or the first four histidines (AAV-GFP-PrP^C^-4HA) abolished the rescue effect (i.e., lower desensitization plateau) observed with the AAV-GFP-PrP^C^ construct.

The first four copper binding motifs on PrP^C^ vary in affinity from femto to nanomolar, with the fifth site (His96) being of lower affinity and being modulated by His111 (see Fig. 1c) [12]. Our data indicate that elimination of the two low affinity copper binding sites (and perhaps associated alterations in affinity for the other sites due to disruptions in cooperativity that is known to occur among these sites [12]) is sufficient to compromise the inhibitory function of PrP^C^ on NMDAR currents. By inference, we thus suggest that copper chelation with BCS may mediate its effect on NMDARs in part by stripping copper from these two lower affinity PrP^C^ copper binding motifs. Our data also show that ablation of the four high affinity sites similarly compromises PrP^C^ function. This then suggests that multiple PrP^C^ copper binding sites participate in the ability of PrP^C^ to inhibit NMDAR activity. Further work with individual substitution of the various histidine residues will be needed to further dissect the copper dependent modulation of NMDARs by PrP^C^, and additional work will be needed to determine if these copper mutants alter NMDAR subunit composition.

## Data Availability

All data generated or analysed during this study are included in this published article.

## Abbreviations

AAV: Adeno-associated virus
NMDAR: N-methyl-d-aspartate receptor
PrP^C^: Cellular prion protein
GFP: Green fluorescent protein
